# The impact of poverty on dog ownership and access to canine rabies vaccination: results from a knowledge, attitudes and practices survey, Uganda 2013

**DOI:** 10.1186/s40249-017-0306-2

**Published:** 2017-06-01

**Authors:** Ryan MacLaren Wallace, Jason Mehal, Yoshinori Nakazawa, Sergio Recuenco, Barnabas Bakamutumaho, Modupe Osinubi, Victor Tugumizemu, Jesse D. Blanton, Amy Gilbert, Joseph Wamala

**Affiliations:** 1 United States Centers for Disease Control and Prevention, Atlanta, GA USA; 2 Uganda Virus Research Institute, Kampala, Uganda; 3 Veterinary Public Health Division, Ministry of Health, Kampala, Uganda; 4 World Health Organization, Kampala, Uganda

**Keywords:** Rabies, Dogs, Vaccination, Poverty, Low-income, Neglected, Africa

## Abstract

**Background:**

Rabies is a neglected disease despite being responsible for more human deaths than any other zoonosis. A lack of adequate human and dog surveillance, resulting in low prioritization, is often blamed for this paradox. Estimation methods are often employed to describe the rabies burden when surveillance data are not available, however these figures are rarely based on country-specific data.

**Methods:**

In 2013 a knowledge, attitudes, and practices survey was conducted in Uganda to understand dog population, rabies vaccination, and human rabies risk factors and improve in-country and regional rabies burden estimates. Poisson and multi-level logistic regression techniques were conducted to estimate the total dog population and vaccination coverage.

**Results:**

Twenty-four villages were selected, of which 798 households completed the survey, representing 4 375 people. Dog owning households represented 12.9% of the population, for which 175 dogs were owned (25 people per dog). A history of vaccination was reported in 55.6% of owned dogs. Poverty and human population density highly correlated with dog ownership, and when accounted for in multi-level regression models, the human to dog ratio fell to 47:1 and the estimated national canine-rabies vaccination coverage fell to 36.1%. This study estimates there are 729 486 owned dogs in Uganda (95% *CI*: 719 919 – 739 053). Ten percent of survey respondents provided care to dogs they did not own, however unowned dog populations were not enumerated in this estimate. 89.8% of Uganda’s human population was estimated to reside in a community that can support enzootic canine rabies transmission.

**Conclusions:**

This study is the first to comprehensively evaluate the effect of poverty on dog ownership in Africa. These results indicate that describing a dog population may not be as simple as applying a human: dog ratio, and factors such as poverty are likely to heavily influence dog ownership and vaccination coverage. These modelled estimates should be confirmed through further field studies, however, if validated, canine rabies elimination through mass vaccination may not be as difficult as previously considered in Uganda. Data derived from this study should be considered to improve models for estimating the in-country and regional rabies burden.

**Electronic supplementary material:**

The online version of this article (doi:10.1186/s40249-017-0306-2) contains supplementary material, which is available to authorized users.

## Multilingual abstracts

Please see Additional file 1 for translations of the abstract into the five official working languages of the United Nations.

## Background

Rabies virus is one of 14 Lyssaviruses, all of which are capable of causing the encephalitic disease known as rabies [1, 2]. While all Lyssaviruses appear to have evolved from a common ancestor that was associated with a chiropteran host, only *rabies virus* appears to have adapted to sustained transmission among terrestrial mammals (primarily *Carnivora* species) [1, 3]. Only *rabies virus* represents a current global health threat; responsible for an estimated 59 000 human deaths and over three billion US dollars in global economic losses annually [4]. The canine rabies virus variant (CRVV) is considered to be responsible for more than 95% of global human rabies deaths. Currently, more than two-thirds of the world’s population resides in a CRVV enzootic country [1, 5].

The CRVV has been successfully eliminated in most developed countries through dog vaccination and targeted public and animal health interventions [6]. Unfortunately, the CRVV remains a significant disease burden in much of sub-Saharan Africa, where an estimated 19 000 rabies deaths occur annually [4]. Despite the advancement of successful interventions, they have not been successfully applied in the majority of sub-Saharan African countries [7]. The neglect of rabies in sub-Saharan Africa is largely attributed to a lack of recognition of rabies as a significant public health threat [8]. This fallacy has been addressed in numerous studies, but the stigma continues to negatively impact rabies control programs in much of the developing world [9–11]. Rabies surveillance is seen as one key activity to improve the recognition of the true public health burden, however, to date, surveillance for rabies is inadequate throughout most of Africa [8, 12, 13].

When surveillance data are lacking, risk models may be useful to describe the estimated burden of animal and human rabies [4, 14]. In Uganda, sparse surveillance data exist for the number of human and canine rabies cases, necessitating the use of modelled estimates to describe the burden of bites (6 602 to 15 778) and human rabies deaths (210 to 592) [15, 16]. Likewise, few studies have captured dog ecology or management information that would be relevant for producing more refined risk models [17]. This lack of country-specific data has resulted in the use of regional and continental data for rabies risk models, which may not be reflective of more refined geographic areas. Therefore, better refined and country-specific estimates of dog densities, rabies vaccination coverage, and barriers to canine vaccination are needed for more effective risk modelling and to inform strategies for rabies control. In the face of high numbers of animal bites and human rabies deaths in Uganda, a knowledge attitudes and practices (KAP) study was conducted for the purpose of enhancing canine rabies control programs in East Africa.

## Methods

A KAP survey on dog ownership and rabies vaccination was conducted among 24 sites in Uganda over a 16 day period in August and September 2013. Five districts geographically distributed from east to west across Uganda were chosen based on two criteria: existence of bite reporting infrastructure and geographical representation of the country. Within each of the five districts, five administrative units were chosen at random utilizing a random number generator, for a total of 25 selected sites. Skip patterns were applied with a target of at least 40 homes per community, while ensuring even distribution. This study, protocol 6312, was approved by the Centers for Disease Control and Prevention’s Human Research Office.

### Survey methods

Surveys were administered to the head of the household or a resident aged ≥ 18 years when the head of the household was not available. One survey was conducted for each participating household. Each house was visited only once. Surveys were conducted in local languages. All survey responses were recorded on handheld personal digital devices (PDAs). Interview locations of participating households were recorded with GPS receivers for mapping purposes. Fingerprints or written informed consent were obtained for all respondents. Consenting respondents received a bar of soap for their participation, in addition to educational materials about rabies prevention and control. Team members were trained in survey and informed consent administration, GPS and PDA use, and project methods 5 days before beginning fieldwork.

### Statistical methods

Data were organized in a three-level hierarchical structure, with households clustered within villages and villages clustered within districts. An unconditional means model was fit, and the likelihood ratio test was used to evaluate the variation of the response between villages and between districts. Multivariable random intercept models were then fit as detailed below to evaluate the effects of household- and village-level characteristics.

### Descriptive model: Dog ownership

Characteristics of dog ownership were examined using logistic regression modelling. Odds ratios (*OR*s) and corresponding 95% confidence intervals were computed; characteristics significantly associated with dog ownership in univariate analysis (*P* < 0.10) were then entered into a multivariable regression model. The statistical significance of each predictor was evaluated using the likelihood ratio test. Backward elimination was conducted and predictor variables were considered significant at *P* < 0.05. Adjusted *OR*s (a*OR*s) and corresponding 95% *CI*s were calculated after controlling for other predictors in the model. Two levels of variables were included in the analysis: Household level characteristics and village-level characteristics (Appendix 1). Household-level characteristics examined as part of the models included: age group of respondent, education level of respondents, household size, years lived in house, livestock value, house building material quality, and rabies knowledge of respondent. House quality was determined by placing an integer value to the construction material of the roof, structure, front door, and windows (Appendix 2). The aggregate of these combined values were used to quantify housing quality. Village-level variables included population density (0 – 100, 101 – 500, 501 – 2 500, and > 2 500 people/km^2^), distance to nearest urban centre (0, 1 – 5 000, 5 001 – 20 000, and > 20 000 meters), and community poverty level (0 – 15, 16 – 35, 36 – 55, and > 55%). Distance to urban centre and population density were highly correlated with each other so only the variable that resulted in the most significant model was chosen.

### Descriptive model: Dog vaccination practices

Characteristics associated with owner-reported previous rabies vaccination among owned dogs were examined using logistic regression modelling. Multivariable regression modelling was conducted as described above. Household-level characteristics examined included the variables listed above in addition to the variables: level of dog care provided, care of community dogs, and rabies education level of respondent. Village-level variables included population density, distance to nearest urban centre, and community poverty level.

### National estimation of Dog population and canine vaccination coverage

Two multivariable random intercept regression models were developed to provide national estimates of the number of owned dogs and the number of vaccinated dogs. For these two models, village-level characteristics were examined by Poisson regression. Village-level characteristics were modelled as continuous variables with an added quadratic term, rather than categorical as used for the descriptive models, to allow for increased precision of national estimates. We obtained a human population map from LandScan (http://web.ornl.gov/sci/landscan/) and a poverty index map from Worldpop (http://www.worldpop.org.uk/) for Uganda, both with spatial resolution of 1 km^2^. All characteristics and relevant interaction terms were entered into multivariable modelling. Backward elimination was performed for model selection as described above.

To estimate the number of owned dogs, a Poisson regression model was developed to estimate the village-level ratio of humans to owned dogs (H:D ratio). These regression coefficients from the final model were multiplied by the human population in 9 km^2^ areas nationwide to produce national dog population estimates. A second model was constructed which estimated the village-average number of vaccinated dogs per person. Regression coefficients from this final model were applied in the manner described above and the estimated number of vaccinated dogs was divided by the estimated number of owned dogs, within the 9 km^2^ cells, to determine the proportion of rabies vaccinated dogs. Three maps were produced for the whole country representing: a) the estimates of number of dogs, b) estimates of vaccinated dogs, and 3) proportion of vaccinated dogs with respect of the total dog population within each cell.

### Estimating human rabies risk

Maintenance of enzootic canine rabies transmission is unlikely in areas with dog densities below 4 dogs/km^2^, and areas where the proportion of vaccinated dogs is 70% or higher [5, 14, 18]. Therefore, based on these premises, we identified human populations within 9 km^2^ areas in which the CRVV is more likely to be maintained (population density ≥ 4 dogs/km^2^ and vaccination below 70%) and thus, represent areas of elevated risk for enzootic rabies transmission (Fig. 3). Human rabies risk was calculated as the rate of unvaccinated dogs per 1 000 human population within the 9 km^2^ areas. This rate was stratified into seven categories to allow for refined estimates of risk.

## Results

Five districts, representing three of Uganda’s four administrative regions, were chosen for inclusion into this study: Kampala, Wakiso, Mbale, Kabarole, and Bundibugyo (Appendix 3). One of the 25 villages could not be surveyed during the study period. A total of 1 000 households were approached, of which 798 completed the survey (range 12–71 surveys per village). The 798 respondents represented a total household study population of 4 375 (5.5 people per household). Dogs were owned by 12.9% of the households (range 0–41.7% per district village), for a total of 175 dogs (H:D ratio 25:1). Population density of the 24 villages surveyed varied greatly (2–2 429 people per km^2^) (Table 1). Village poverty levels also varied greatly (5.4–72.6% of residents in poverty). The average poverty level (measured as percent of people living below the international poverty line of US $1.25 per day) among the villages in this study was 45%, compared to a Ugandan national average of 38% (http://www.unicef.org/infobycountry/uganda_statistics.html).

**Table 1:** Comparison of village characteristics from a survey assessing dog ownership practices: Uganda, 2013

District	Village ID	Population Density (km^2^)	Distance to Urban Centre (km)	Percent Below Poverty	Households Interviewed	Study Population	Number of Dogs	Dog Owning HH	Dogs/HH Observed	People per Dog
Kampala	AZ1	1 401	0	5.7%	38	177	0	0 (0.0%)	0.00	-
	MU1	34	2	20.6%	12	57	8	5 (41.7%)	0.66	7.1
	KZ1	286	0	20.8%	19	108	3	1 (5.3%)	0.16	36.0
	KE1	2 429	0	14.0%	22	94	0	0 (0.0%)	0.00	-
	CZ1	433	0	19.9%	19	109	18	3 (15.8%)	0.95	6.1
Wakiso	NC2	158	3	29.7%	48	178	6	4 (8.3%)	0.13	29.7
	BU2	31	15	40.0%	34	153	9	8 (23.5%)	0.26	17.0
	BG2	3	14	39.7%	17	66	3	3 (17.6%)	0.18	22.0
	MB2	41	14	39.2%	29	156	5	5 (17.2%)	0.17	31.2
	KI2	41	10	37.9%	29	147	3	3 (10.3%)	0.10	49.0
Mbale	BA3	34	2	59.5%	60	374	2	2 (3.30%)	0.03	187.0
	KA3	633	0	52.9%	33	190	11	4 (12.1%)	0.33	17.3
	MB3	49	4	61.5%	33	201	4	3 (9.1%)	0.12	50.3
	BU3	57	18	56.0%	52	273	2	2 (3.8%)	0.04	136.5
	NM3	441	0	52.9%	71	357	2	2 (2.8%)	0.03	178.5
Kabarole	RW4	43	25	56.4%	23	163	9	6 (26.1%)	0.39	18.1
	NY4	447	1	49.2%	37	209	13	9 (24.3%)	0.35	16.1
	KK4	12	11	52.0%	46	259	31	16 (34.8%)	0.67	8.4
	BU4	10	1	52.7%	31	159	20	11 (35.5%)	0.65	8.0
	KI4	226	1	51.4%	30	149	12	6 (20.0%)	0.40	12.4
Bundibugio	KY5	3	33	74.5%	28	214	1	1 (3.6%)	0.04	214
	BB5	36	31	63.6%	32	231	3	3 (9.4%)	0.09	77.0
	BG5	2	21	60.0%	37	234	10	6 (16.2%)	0.27	23.4
	HK5	74	26	56.3%	18	117	0	0 (0.0%)	0.00	-
	TOTAL	288	9.7	44.4%	798	4 375	175	103 (12.9%)	0.22	25.0

### Attitudes towards Dog ownership and rabies vaccination

The lowest rates of dog ownership and dog densities were observed within villages with the highest poverty levels, ≥ 56% (58.3 people per dog vs average 25.0) (Table 2). The annual canine death rate was 101 deaths per 1 000 dogs (10.1%). The most commonly reported cause of dog death was disease, which was implicated in 39.3% of deaths, followed by injury (36.5%) and unknown causes (15.7%). Disease deaths were more frequently reported among dogs from villages with poverty levels > 35% (42.9% and 52.2% of dog deaths in the two highest poverty categories).

**Table 2:** health indicators for owned dogs by community poverty level, Uganda 2013

	Village Poverty Level				
	0–19%	20–35%	36–55%	≥56%	Total
	*n* (%)	*n* (%)	*n* (%)	*n* (%)	*n* (%)
Number of dogs^a^	18 (10.3%)	17 (9.7%)	109 (62.3%)	31 (17.7%)	175 (100%)
Study population^a^	380 (8.7%)	343 (7.8%)	1 845 (42.2%)	1 807 (41.3%)	4 375 (100%)
Persons per dog	21.1	20.2	16.9	58.3	25.0 *
Dog Owning Households	3 of 79 (3.8%)	10 of 79 (12.7%)	67 of 357 (18.8%)	23 of 283 (8.1%)	103 of 798 (12.9%) *
Dogs per Dog Owning Household	6	1.7	1.6	1.3	1.7
Modelled Dogs per km^2^	485	72	49	19	96
Average Dog Age (95% *CI*)	2.3 (1.1–3.5)	2.9 (1.8–3.9)	2.8 (2.1–3.0)	1.9 (1.5–2.3)	2.4 (2.0–2.8)
Dogs with history of rabies vaccination	18 (100.0%)	12 (70.6%)	56 (51.4%)	13 (41.9%)	99 (56.6%) *
Suspected Rabies Dog Deaths, Past 5 years	0	0	5	4	9
Rabies Rate (annual, per 1 000 dogs)^b^	0	0	9.2	8.2	5.1
Households with dog deaths past 5 years	7 (8.9%)	11 (13.9%)	49 (13.7%)	31 (11.0%)	98 (12.3%)
Number of Dog Deaths, past 5 years	7 (100%)	34 (100%)	70 (100%)	67 (100%)	178 (100%)
Injury	3 (42.9%)	16 (47.1%)	24 (34.3%)	22 (32.8%)	65 (36.5%)
Disease	0 (0.0%)	5 (14.7%)	30 (42.9%)	35 (52.2%)	70 (39.3%)
Poison	0 (0.0%)	2 (5.9%)	2 (2.9%)	7 (10.4%)	11 (6.2%)
Natural Causes	0 (0.0%)	0 (0.0%)	4 (5.7%)	0 (0.0%)	4 (2.2%)
Unknown Causes	4 (57.1%)	11 (32.4%)	10 (14.3%)	3 (4.5%)	28 (15.7%)
Dog Death Rate (annual, per 1 000 dogs)^c^	56	133	78	137	101

^a^row percentage

^b^Rabies suspected death: dogs that died shortly after displaying at least two of the following symptoms: aggression, biting, hypersalivation, paralysis, lethargy. Canine rabies rates was calculated as: ((Rabies Deaths _n_/(Alive dogs _n_ + Dead Dogs _n_))/5 years) × 1 000 dogs

^c^Dog death rate: ((Dead Dogs _n_/(Alive dogs _n_ + Dead Dogs _n_))/5 years) × 1 000 dogs

*Indicates Cochran Chi Square *P* value < 0.01

Of the 175 owned dogs identified in this study, 99 had a reported history of rabies vaccination (56.6%) (Table 2). Dogs were more likely to have a history of vaccination when they resided in low poverty villages (100, 70.6, 13.7, and 11.0%, respective to low-high poverty rate). Suspected rabies deaths among dogs were reported from the two highest poverty categories (*n* = 5 and 4, respectively) but none were reported from the two lowest poverty categories. The rate of suspected canine rabies among dogs in the study population was 5.1 per 1 000 dogs (range 0–9.2).

Owners reported that 31.4% of dogs were always allowed to roam freely and 21.7% were always confined to the owner’s property, 4% of owners reported an unknown confinement status; the remaining dogs were intermittently free-roaming (Table 3). Overall, 74.3% of dogs were allowed to roam freely to some degree. Free roaming dogs were more frequently reported among villages in the two highest poverty classifications (78.0 and 74.2% dogs free-roaming) compared to villages in the two lowest poverty classifications (61.1 and 64.7%). The majority of dog owners provided their dog’s food and water (95.1 and 81.6%), however fewer than half of owners provided their dogs with veterinary care or shelter (43.7 and 37.9%).

**Table 3:** Characteristics of Dog Ownership Practices by Community Poverty Level, Uganda, 2013

	Community poverty level					
Poverty classification	0–15%	16–35%	36–55%	>55%	Total	Cochran *P* value
	n (%)	n (%)	n (%)	n (%)	n (%)	
Number of dogs	18	17	109	31	175	
Number of people	380	343	1 846	1 807	4 376	
Number of households	79	79	357	283	798	
Dog owning households	3 (3.8%)	10 (12.7%)	67 (18.8%)	23 (8.1%)	103 (12.9%)	
How Often Are Dogs Allowed to Roam Freely?						<0.01
Always	0 (0.0%)	4 (23.5%)	38 (34.9%)	13 (41.9%)	55 (31.4%)	
Occasionally	0 (0.0%)	3 (17.6%)	21 (19.3%)	2 (6.5%)	26 (14.9%)	
Infrequently	11 (61.1%)	4 (23.5%)	26 (23.9%)	8 (25.8%)	49 (28.0%)	
Never	0 (0.0%)	6 (35.3%)	24 (22.0%)	8 (25.8%)	38 (21.7%)	
Unknown	7 (38.9%)	0 (0.0%)	0 (0.0%)	0 (0.0%)	7 (4.0%)	
Number of dogs allowed to roam freely	11 (61.1%)	11 (64.7%)	85 (78.0%)	23 (74.2%)	130 (74.3%)	0.36
Level of care households provided for dogs^a^						
None	0 (0.0%)	0 (0.0%)	2 (3.0%)	0 (0.0%)	2 (1.9%)	0.78
Food	2 (66.7%)	9 (90.0%)	65 (97.0%)	22 (95.7%)	98 (95.1%)	0.10
Water	2 (66.7%)	9 (90.0%)	55 (82.1%)	18 (78.3%)	84 (81.6%)	0.78
Shelter	2 (66.7%)	6 (60.0%)	24 (35.8%)	7 (30.4%)	39 (37.9%)	0.28
Veterinary Care	2 (66.7%)	9 (90.0%)	27 (40.3%)	7 (30.4%)	45 (43.7%)	0.01
Households with Unvaccinated Dogs	0 (0.0%)	3 (30.0%)	38 (56.7%)	13 (56.5%)	54 (52.4%)	0.11
Reason Owners did not Vaccinated Dogs^b^						
Dog is too young	0 (0.0%)	1 (33.3%)	7 (18.4%)	1 (7.7%)	9 (16.7%)	
No time	0 (0.0%)	0 (0.0%)	1 (2.6%)	2 (15.4%)	3 (5.6%)	
No money to buy vaccine	0 (0.0%)	1 (33.3%)	4 (10.5%)	0 (0.0%)	5 (9.3%)	
No vaccine available	0 (0.0%)	0 (0.0%)	20 (52.6%)	7 (53.8%)	27 (50.0%)	
Government vaccination did not occur	0 (0.0%)	0 (0.0%)	7 (18.4%)	3 (23.1%)	10 (18.5%)	
No need to vaccinate/Did not know needed to vax	0 (0.0%)	1 (33.3%)	0 (0.0%)	2 (15.4%)	3 (5.6%)	
Unknown reason	0 (0.0%)	1 (33.3%)	0 (0.0%)	0 (0.0%)	1 (1.9%)	
Households providing care to community dogs	2 (2.5%)	5 (6.3%)	44 (12.3%)	28 (9.9%)	79 (9.9%)	0.04
Number of community dogs cared for	11	12	205	111	339	
Level of care provided to community dogs^b^						
Food	2 (2.5%)	4 (5.1%)	43 (12.0%)	29 (10.2%)	78 (9.8%)	
Water	0 (0.0%)	2 (2.5%)	17 (4.8%)	10 (3.5%)	29 (3.6%)	
Shelter	0 (0.0%)	1 (1.3%)	0 (0.0%)	1 (0.4%)	2 (0.3%)	
Veterinary Care	0 (0.0%)	0 (0.0%)	0 (0.0%)	1 (0.4%)	1 (0.1%)	
Other	0 (0.0%)	1 (1.3%)	1 (0.3%)	0 (0.0%)	2 (0.3%)	

^a^variables are not mutually exclusive, therefore a Cochran *p*-value can be calculated for each row

^b^cell values are too small to calculate a Cochran *P* value

On average 52.4% of dog-owning households reported owning at least one dog that was not vaccinated against rabies (range 0–56.7%) (Table 3). The most commonly reported response for owning an unvaccinated dog was that “no vaccine was available” (50.0%), followed by “the government vaccination did not occur” (18.5%). Vaccine availability through the government and other sources was reported as a barrier to vaccination among dog owners residing in the two highest poverty categories (69 and 76.9%).

Overall, 79 of the 778 households reported that they provided some level of care to dogs which they did not own (10.3%) (Table 3). Providing care to community dogs was more frequently reported in higher poverty villages. The most common care provided to community dogs was food (9.8% of survey respondents), followed by water (3.6%). Veterinary care and shelter were almost never provided to dogs which were not owned by the survey respondents (0.1 and 0.3%, respectively). The number of unowned dogs in these villages could not be ascertained from the study design.

### Multivariable logistic regression of Dog ownership and vaccination practices

The variables ‘household size’, ‘livestock value’, ‘home building material quality’, and ‘village poverty level’ were all significant in multivariable analysis (Table 4). Households with more than seven residents had 3.3 greater odds of owning a dog. Households which owned $1–$199 USD in livestock value were at 4.3 greater odds of owning a dog compared to households with no livestock value. Households with more than $1 000 USD in livestock value had the greatest odds of owning at least one dog compared to households with no livestock value (a*OR* = 19.6, 95% *CI*: 7.9–48.7). Households which were made of high quality building materials were at 2.6 greater odds of owning a dog compared to households consisting of low quality building materials (95% *CI*: 1.3–5.2). Households residing in a village with an average poverty level of 16–35% had 7.7 greater odds of owning a dog compared to households in villages of the lowest poverty category (95% *CI*: 1.5 – 40.0). Household dog ownership was not significantly associated with the two highest poverty classifications.

**Table 4:** Characteristics Associated with Household Dog Ownership by Univariate and Multivariable Methods, Uganda 2013

Characteristic		Do Not Own a Dog	Own at Least One Dog	Mean number of dogs owned	Unadjusted Odds Ratio	Adjusted Odds Ratio
		*n* (column %)	*n* (column %)	mean (SE)	*OR* (95% *CI*)	a*OR* (95% *CI*)
Demographic						
Household size	1–2	111 (16.0)	6 (5.8)	0.06 (0.27)	Reference	Reference
	3–4	214 (30.8)	19 (18.5)	0.12 (0.42)	1.9 (0.72–5.04)	1.41 (0.58–4.15)
	5–6	166 (23.9)	23 (22.3)	0.29 (1.11)	3.04 (1.15–8)	2.06 (0.70–6.01)
	7 +	204 (29.4)	55 (53.4)	0.33 (0.79)	6.57 (2.61–16.54)	3.26 (1.16–9.18)
Years in house	0–2 years	175 (25.2)	14 (13.6)	0.17 (0.87)	Reference	
	3 + yrs	520 (74.8)	89 (86.4)	0.23 (0.72)	2.25 (1.22–4.16)	
Village population density (people/km2)	0–100	133 (19.1)	41 (39.8)	0.40 (0.92)	Reference	
	101–500	256 (36.8)	33 (32.0)	0.14 (0.44)	0.73 (0.41–1.31)	
	501–2 500	104 (15.0)	11 (10.7)	0.18 (0.72)	0.36 (0.17–0.76)	
	2 501 +	202 (29.1)	18 (17.5)	0.20 (0.92)	0.44 (0.21–0.92)	
Distance to nearest urban centre (km)	0	199 (28.6)	15 (14.6)	0.20 (0.94)	Reference	
	1–5 000	204 (29.4)	35 (34.0)	0.24 (0.70)	1.2 (0.35–4.12)	
	5 001–20 000	170 (24.5)	37 (35.9)	0.26 (0.74)	1.74 (0.51–5.96)	
	20 001 +	122 (17.6)	16 (15.5)	0.17 (0.52)	1.07 (0.26–4.35)	
Economics						
Owned livestock value (USD)	$0	291 (41.9)	10 (9.7)	0.10 (0.72)	Reference	Reference
	$1–$199	192 (27.6)	20 (19.4)	0.11 (0.39)	3.94 (1.68–9.26)	4.33 (1.71–10.93)
	$200–$999	145 (20.9)	32 (31.1)	0.31 (0.85)	8.86 (3.77–20.82)	9.81 (3.87–24.88)
	$1 000 +	67 (9.6)	41 (39.8)	0.62 (1.05)	20.77 (8.93–48.3)	19.61 (7.90–48.68)
Home building material quality	High	148 (21.4)	38 (36.9)	0.41 (1.16)	3.2 (1.73–5.89)	2.59 (1.30–5.17)
	Medium	174 (25.1)	20 (19.4)	0.19 (0.70)	1.02 (0.55–1.9)	0.79 (0.40–1.55)
	Low	371 (53.5)	45 (43.7)	0.15 (0.50)	Reference	Reference
Village poverty level	0–15%	76 (10.9)	3 (2.9)	0.23 (1.29)	Reference	Reference
	16–35%	69 (9.9)	10 (9.7)	0.22 (0.63)	4.76 (1.01–22.41)	7.65 (1.46–40.04)
	36–55%	290 (41.7)	67 (65.1)	0.31 (0.82)	7.04 (1.33–37.41)	4.66 (0.79–27.37)
	56% +	260 (37.4)	23 (22.3)	0.11 (0.41)	4.13 (0.62–27.53)	2.28 (0.32–16.47)

Among the variables considered for multivariable logistic regression to predict ownership of a vaccinated dog, only village population density, age of the dog, and the confinement of the dog remained in the adjusted model (Table 5). Dogs residing in villages with a human population density per km^2^ greater than 2 501 were at 7.9 greater odds of being vaccinated against rabies compared to dogs residing in villages with a human population density below 100 people per km^2^ (95% *CI*: 2.5–24.8). All dogs older than 1 year of age had greater odds of being vaccinated against rabies, compared to dogs less than 1 year of age. Dogs which were always confined to the owner’s control had significantly greater odds of being vaccinated compared to dogs that were always allowed to roam freely (a*OR* = 25.4, 95% *CI*: 4.9–132.9). Economic indicators were not significantly associated with household dog vaccination in the adjusted model.

**Table 5:** Characteristics associated with canine vaccination rates by univariate and multivariable methods, Uganda 2013

		Vaccinated Dogs *n* (%)	Not Vaccinated *n* (%)	Odds Ratio (95% *CI*)	*P* value	Adjusted Odds Ratio (95% *CI*)
Demographic						
Household size	1–3	11 (50.0)	11 (50.0)	Reference		
	4–6	43 (64.2)	24 (35.8)	1.8 (0.7–4.7)	0.20	
	7–9	34 (53.1)	30 (46.9)	1.1 (0.4–3.0)	0.80	
	>9	11 (50.0)	11 (50.0)	1.0 (0.3–3.3)	1.00	
Village population density (km^2^)	0–100	30 (43.5)	39 (56.5)	Reference		Reference
	101–500	16 (39.0)	25 (61.0)	0.8 (0.4–1.8)	0.65	0.4 (0.1–1.1)
	501–2 500	17 (81.0)	4 (19.0)	5.5 (1.7–18.1)	<0.01	3.3 (0.8–13.2)
	2 501 +	36 (81.8)	8 (18.2)	5.9 (2.4–14.1)	<0.01	7.9 (2.5–24.8)
Distance to urban center (km)	>10 000	30 (39.5)	46 (60.5)	Reference		
	1–10 000	36 (63.2)	21 (36.8)	2.6 (1.3–5.3)	<0.01	
	0	33 (78.6)	9 (21.4)	5.6 (2.4–13.4)	<0.01	
Economic						
Owned livestock value (USD)	0	24 (82.8)	5 (17.2)	6.4 (1.9–21.7)	<0.01	
	1–200	12 (42.9)	16 (57.1)	Reference		
	201–500	8 (42.1)	11 (57.9)	1.0 (0.3–3.2)	1.00	
	501–2 000	34 (50.8)	33 (49.2)	1.4 (0.6–3.3)	0.49	
	>2 000	21 (65.6)	11 (34.4)	2.5 (0.9–7.2)	0.08	
Home building material quality	Low	3 (21.4)	11 (78.6)	Reference		
	Medium	25 (43.1)	33 (56.9)	2.8 (0.7–11.0)	0.13	
	High	71 (68.9)	32 (31.1)	8.1 (2.1–31.2)	<0.01	
Village poverty level	0–15%	18 (100)	0 (0.0)	50.7 (2.8–917.2)	<0.01	
	16–35%	12 (70.6)	5 (29.4)	3.2 (0.9–11.8)	0.06	
	36–55%	56 (51.4)	53 (48.6)	1.5 (0.7–3.3)	0.36	
	56% +	13 (41.9)	18 (58.1)	Reference		
Animal Care						
Care provided to owned dogs	No care	11 (44.0)	14 (56.0)	Reference		
	Minimal	16 (39.0)	25 (61.0)	0.8 (0.3–2.3)	0.44	
	Moderate	40 (54.8)	33 (45.2)	1.5 (0.6–4.3)	0.24	
	High	32 (88.9)	4 (11.1)	10.2 (2.8–37.6)	<0.01	
Dog Age (years	0–1	34 (38.6)	54 (61.4)	Reference		Reference
	>1–3	21 (70.0)	9 (30.0)	3.7 (1.5–9.0)	<0.01	12.3 (4.0–38.2)
	>3–5	23 (71.9)	9 (28.1)	4.1 (1.7–9.8)	<0.01	8.5 (2.8–25.6)
	>5	21 (84.0)	4 (16.0)	8.3 (2.6–26.4)	<0.01	16.9 (4.2–67.6)
Dog Confinement	Never	24 (43.6)	31 (56.4)	Reference		Reference
	Rarely Confined	14 (53.9)	12 (46.1)	1.5 (0.6–3.9)	0.2	1.2 (0.3–4.4)
	Frequently Confined	31 (63.3)	18 (36.7)	2.2 (1.0–4.9)	0.02	1.8 (0.5–6.3)
	Always	23 (60.5)	15 (39.5)	2.0 (0.8–4.6)	0.06	25.4 (4.9–132.9)

### National estimations of owned dogs and rabies vaccination coverage

**Fig. 1:**
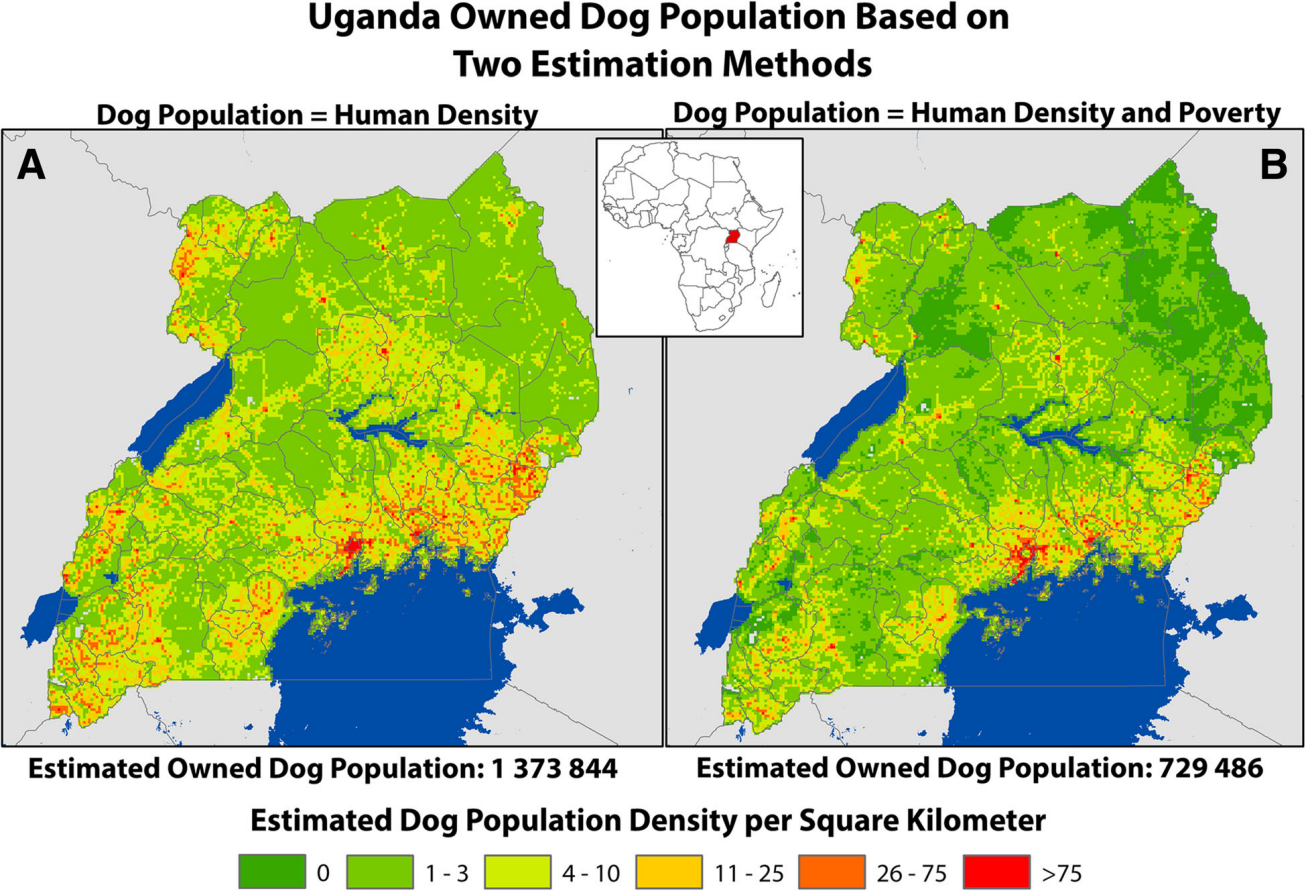
Comparison of Two Methods for Estimating the Density and Distribution of Owned Dog Populations, Uganda 2013. **a** Estimate of dog density based on constant ratio of dogs to humans based on findings from study: 1 dog for every 25 people. **b** Estimate of dog density based on multivariable random intercept regression models: Dog Population = ˗ 3.33 + (˗ 0.002 × human population density) + (˗ 2.27 × village poverty level) + (0.12 × human population density × village poverty level)

Multilevel logistic regression for the prediction of dog population had two significant predictor variables, human population density and village poverty level, as well as the interaction term of these two variables (Fig. 1). When the regression model was extrapolated nationally to each 9 km^2^ cell in Uganda, a predicted total of 729 486 owned dogs were estimated for Uganda (95% *CI*: 719 919–739 053). Given a human population of 34 346 101, the national average H:D ratio was 47:1.

$$ \mathrm{Dog}\ \mathrm{Population} = - 3.33 + \left( - 0.002 \times \mathrm{human}\ \mathrm{population}\ \mathrm{density}\right) + \left( - 2.27 \times \mathrm{village}\ \mathrm{poverty}\ \mathrm{level}\right) + \left(0.12 \times \mathrm{human}\ \mathrm{population}\ \mathrm{density} \times \mathrm{village}\ \mathrm{poverty}\ \mathrm{level}\right) $$

**Fig. 2:**
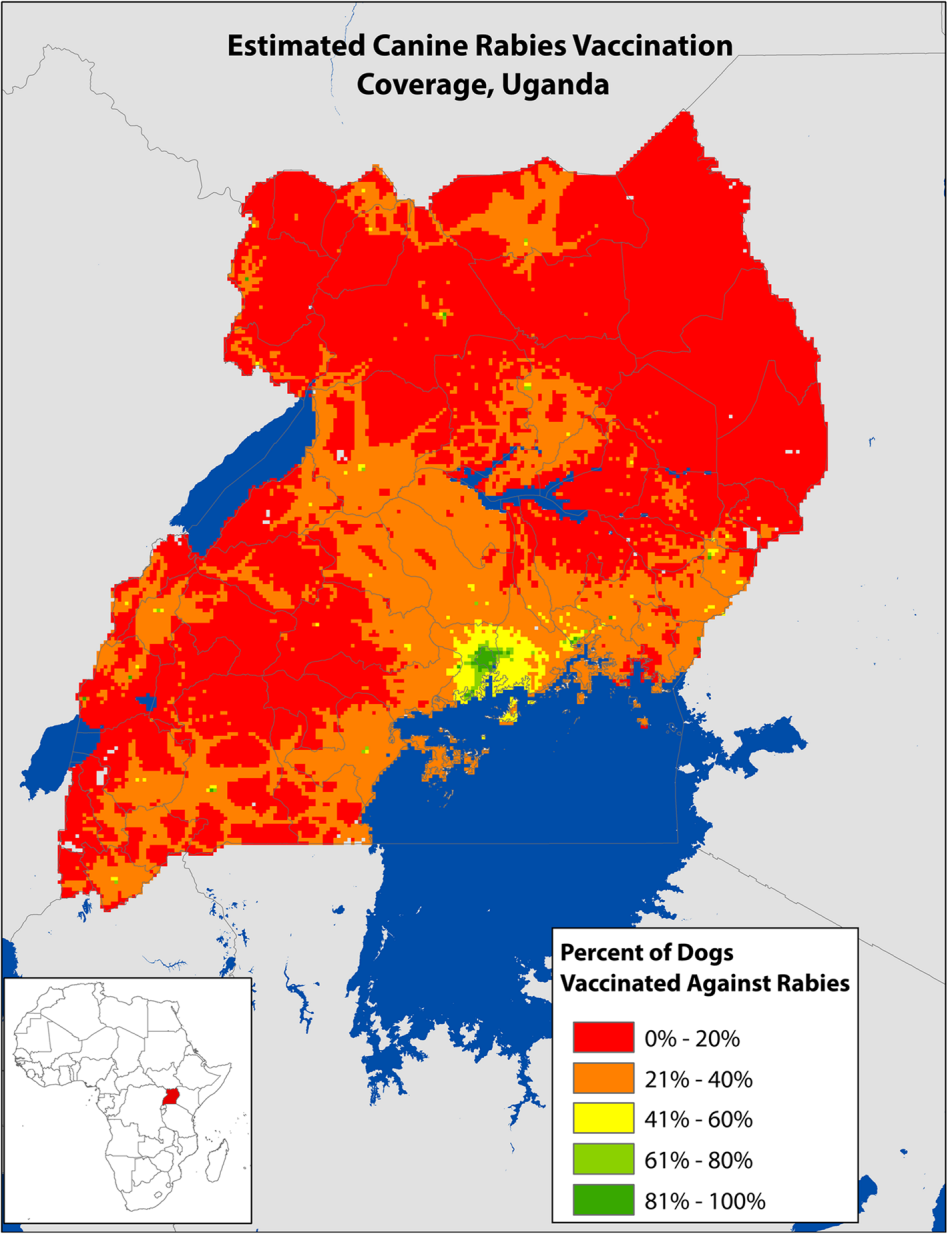
Estimated Canine Rabies Vaccination Coverage, Uganda 2013. *Estimation based on modelled estimates: Dog Vaccination = Population Density + Poverty Level + (Population Density × Poverty Level). ** National canine rabies vaccination rate estimated to be: 35% with high levels in Kampala and low levels in rural areas

Multilevel logistic regression for the prediction of the rabies vaccinated dog population had two significant predictor variables: human population density and village poverty level, as well as the interaction term of these two variables (Fig. 2). When the regression model was extrapolated nationally to each 9 km^2^ cell in Uganda, a predicted total of 257 995 owned, vaccinated dogs are expected to be present in Uganda, for an estimated national canine rabies vaccination rate of 35.4%.

$$ \mathrm{Vaccinated}\ \mathrm{Dogs} = - 4.03 + \left(0.02 \times \mathrm{human}\ \mathrm{population}\ \mathrm{density}\right) + \left( - 4.22 \times \mathrm{village}\ \mathrm{poverty}\ \mathrm{level}\right) + \left(0.17 \times \mathrm{human}\ \mathrm{population}\ \mathrm{density} \times \mathrm{village}\ \mathrm{poverty}\ \mathrm{level}\right) $$

### Estimating human rabies risk

**Fig. 3:**
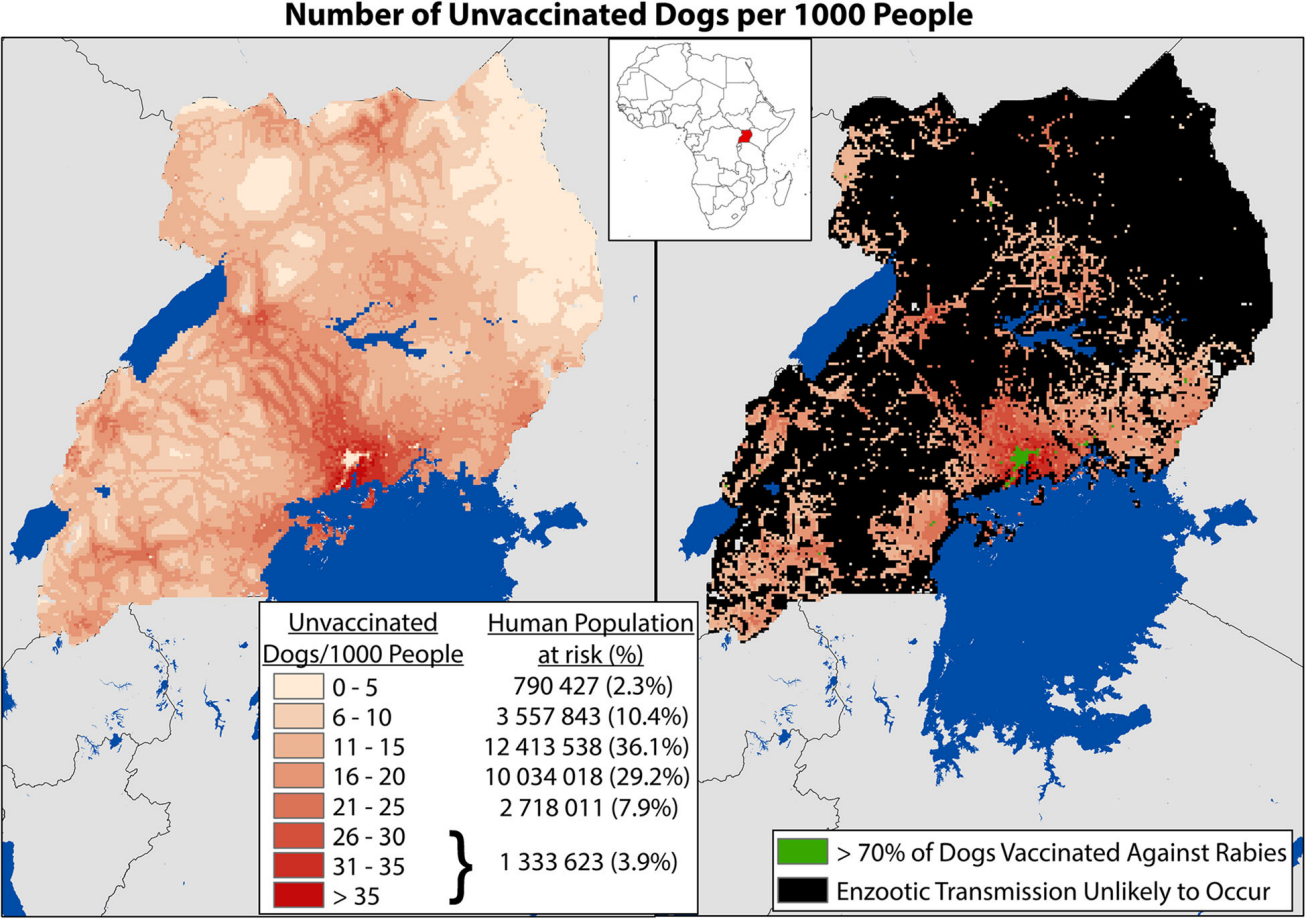
Risk of Canine Rabies Transmission as Displayed by the Number of Unvaccinated Dogs per 1 000 Human Population, Uganda 2013. *Model estimated were used to predict the number of unvaccinated dogs per 1 000 human population. Areas with vaccination coverage > 70% are identified in *grey*, as enzootic transmission is not thought to occur at these vaccination levels. Areas with fewer than 4 dogs per square kilometre are identified in *black*, as the dog population density may be too low to support enzootic transmission of the virus. However, areas in *black* are still susceptible to epizootic events when rabid animals are introduced to the community, such as the case with importing dogs from other rabies enzootic communities. The areas remaining in *red* are places with estimated large populations of both people and unvaccinated dogs, representing a greater risk for dog to human rabies transmission events

Based on modelled estimates, only 9.8% of the Ugandan population resides in an area in which over 70% of the dogs are expected to have had any history of vaccination against rabies (Fig. 3). An additional 206 916 Ugandans are estimated to reside in areas where dog population densities are below 4 dogs/km^2^. The remaining 30 847 460 Ugandans (89.8%) reside in areas where there is the theoretical possibility for enzootic canine rabies transmission (Fig. 3).

## Discussion

Understanding the distribution and ecology of dog populations is critical for the planning and implementation of effective canine rabies control strategies. In addition, this knowledge can aid development of more accurate estimation methods for the burden of animal and human rabies deaths. The latter is often necessary in many developing countries where surveillance efforts are inadequate to accurately describe disease burden. This study represents one of the most comprehensive attempts to characterize the dog population and rabies risk in Uganda.

### Dog ownership and poverty

Accurate estimates of dog populations are critical for the planning of mass rabies vaccination programs. Most studies calculate dog populations as a factor of human population or land mass (km^2^) [17]. To our knowledge, this is the first study to conduct a comprehensive analysis of the impact of poverty on dog ownership, and our findings support that this interaction greatly effects the total estimated dog population. The rate of dogs per person for the African continent have been estimated at 21:1 (urban) and 7:1 (rural) [14, 17]. The unadjusted H:D ratio in this study is in line with these regional and continental estimates (25 people per dog); and applying this rate to Uganda’s population of 36 million people, results in a national dog population of 1.3 million. However, there was a clear and strong association between dog ownership and village poverty identified in this study. When considering the effect of poverty, the adjusted estimated H:D ratio was nearly 2-fold higher (47:1) compared to the unadjusted estimates.

The model developed as part of this study suggests that areas with high poverty/low population density owned fewer dogs (i.e. poor, rural settings). Likewise, areas with low poverty/high population density owned fewer dogs (i.e. affluent, urban settings). However, areas with high poverty and high population density had a positive correlation with dog ownership (i.e. poor, urban settings). While surprising that the modelled estimates are much lower than the non-adjusted H:D ratio, there are several other African countries that have reported similar findings through differing population estimation methods [19, 20]. These results suggest that modelling of dog populations is likely not as simple as applying a standardized rate to a human population, and that poverty levels should be considered a potential confounder in the relationship between man and dog.

There are several methods described for estimating dog populations, including street counting, capture-recapture, registry records, and KAP surveys. However, none of these methods are capable of accurately capturing all types of dogs (owned, community owned, and feral). For example, counting methods rely on the dog being visible to the counter and thereby accurately estimates only the free-roaming dog population in a community, but neglect the proportion of dogs that remain within the home. Registries and KAP studies rely on the self-reported ‘ownership’ of the dogs. Therefore, in these methods, community-owned and feral dogs may not be accurately counted. However, there are two reasons why this may not be a significant limitation under certain scenarios. If the goal of the dog population estimation is to inform a national vaccination strategy which only utilizes point-source or door-to-door vaccination, where only the owned dog population is reachable, then a KAP survey method would provide accurate data for planning such campaigns. Additionally, if there are relatively few community dogs within the population, then KAP survey methods may also be accurate.

A primary goal of a rabies vaccination program should be to describe the ownership status (owned, community, feral) and the confinement status (confined, semi-confined, free roaming) of the dog population. Most studies have shown agreement that feral dogs, which are dogs that survive on no directly provided human support, are rare (less than 1% of a dog population in the majority of settings) [21, 22]. However, the significance of community dogs on the overall dog population can vary greatly and often depends on the cultural and economic situation. The method used in this study only accurately accounts for the ‘owned dog’ population. Survey respondents were asked about their interactions with community dogs, of which 10% said they provided some level of care. Unfortunately, it is impossible to determine the degree to which including these dogs could impact the total dog population estimation, as community dogs, by definition, receive care from numerous sources and many homes likely reported overlapping dogs in this count. A simple sensitivity analysis, in which our modelled estimate is considered the lowest population, and a 10% increase due to community dogs would be the highest estimate, still is vastly lower than the unadjusted H:D ratio and regional H:D ratios that would be applied to Uganda if poverty was not considered (719 919–812 958 dogs vs 1 300 000 dogs).

There are potentially harmful consequences from under-estimating a dog population, such as the under vaccination of dogs which may increase the rabies burden in a community or lead to an increase in outbreaks [23, 24]. Therefore, this newly developed model for dog population estimation as a function of human population density and poverty should be validated through field studies utilizing alternative methods. If it is shown that the model developed in this analysis is accurate, this may provide added incentive for governments to increase vaccination programs, as the target of 70% would be more easily achieved.

### Dog vaccination rates and poverty

Canine vaccination rates in the villages assessed through this study varied greatly, from a low of 0% vaccinated to a high of 100% vaccinated. Overall, the unadjusted canine vaccination rate in this study was surprisingly high (56.6%), yet still below the target for effective herd immunity (70%). However, on closer review this vaccination coverage rate was heavily biased by the poverty level of the community, and when accounting for this bias, the national canine rabies vaccination rate was down-adjusted to 35.4%.

A recent study on the global burden of rabies estimated 10% vaccination coverage for dogs in Uganda, far below the reported and modelled values found in this study [4]. This study design asked only if the dog had ever been vaccinated against rabies, and did not record when or how frequently the animal had been vaccinated. Therefore, it is likely that a proportion of these dogs would not qualify as ‘properly vaccinated’ by WHO standards (having received at least 2 vaccinations during lifetime) [5]. Additionally, this study does not reflect the vaccination practices of community dogs. Fewer than 2% of survey respondents indicated that they provided veterinary care to community dogs, so in places where community dogs make a significant proportion of the population the vaccination rate will likely be decreased. As a result, the level of rabies herd immunity in Uganda is likely to be lower than reported here. In reality, the true population-level vaccination coverage for rabies in Ugandan dogs likely lies between the previously estimated 10% and the value identified in this study.

Barriers to vaccination were frequently reported among study participants, particularly in villages with higher rates of poverty. Encouraging, however, was the finding that all dogs in higher income villages were reportedly vaccinated against rabies, an indication that successful vaccination strategies can be, and have been, implemented. The most commonly reported barrier to canine rabies vaccination was a lack of ability for the owner to procure vaccine, both privately and through government campaigns. This likely reflects the current situation in Uganda and many developing countries, in which canine rabies vaccine is typically available to dog owners only during periodic, nationally supported, vaccination campaigns. During years in which these national campaigns do not reach villages or in which not enough vaccines are procured, there are no other options. These findings, while not surprising, should emphasize the important public service role that governments must play to realize successful rabies vaccination programs.

A critical ecological measure that can help predict rabies vaccination success is the population turnover rate among dogs. Communities with high dog population turnover will require more frequent and intensive canine vaccination campaigns [23]. For example, a community with 70% vaccination coverage, but a 50% annual death rate among their dogs would see the level of herd immunity drop to 53% after only 6 months and 35% after one year. Justifiably, monitoring the overall health of the dog population is an important evaluation measure for rabies control programs [25]. In this study a canine death rate of 10% was identified, but ranged from 5% in low poverty areas to 14% in high poverty areas. These figures are actually much lower than other published studies in developing countries, which have shown population turnover rates reaching greater than 30% [26, 27]. The most common causes of death were injury and disease, both preventable through responsible dog ownership and provision of veterinary care. Improving dog ownership practices through promotion of animal welfare education, leash laws, and reliable access to veterinary care could have positive impacts on the canine vaccination rates and directly benefit humans through decreases in bite events and rabies deaths.

The benefits of canine rabies vaccination were displayed in this study, where it was shown that in areas of high vaccination coverage there were no owner-reported incidents of dog deaths which were consistent with rabies. However, among high poverty villages the canine rabies vaccination rates were less than 15%, and the rate of dog deaths suspected to be rabies were much higher. Of note, canine distemper virus may present with signs similar to rabies, and is common in Uganda, therefore the rate of suspected rabies may be lower than what was estimated here [28].

By understanding the dog ownership characteristics in representative communities, one can then extrapolate the information to larger areas and thereby make more informed national rabies control policies. In this study we quantified national dog densities utilizing country-specific data and obtained drastically different results from Knobel et al. [14], who utilized regional and global data (Fig. 1). It is apparent in Fig. 1 that a large portion of Uganda has very low expected dog densities. Recent publications have suggested that rabies cannot remain enzootic in areas for which the dog density is below approximately 4 per km^2^, identified in black in Fig. 3 [14, 18, 22]. While enzootic transmission may be unlikely, rabies outbreaks in these communities are still possible if vaccination rates are low and dogs from enzootic areas are introduced, a practice commonly documented in many canine rabies endemic countries [25]. By this logic, targeted vaccination of the surrounding higher dog-density communities may have regional impact on the rate of rabies, and may represent a more cost-effective method of eliminating the disease in dogs. Where resources are available, mass vaccination of all dogs is recommended, however where resources are limited they should be used to maximum efficiency. In these situations, modelling of dog populations and identification of areas in which dog vaccination would provide the most benefit to society should be conducted.

### Estimating the human rabies risk

There are numerous factors that must be taken into account when trying to accurately predict the risk of human rabies. The data collected in this study should be used to refine these complex estimation models. However, a more simple approach to estimating human rabies risk was undertaken here, where the modelled outputs of canine vaccination coverage and dog density were used to approximate the areas in Uganda in which more than 70% of dogs were likely vaccinated against rabies and areas where fewer than 4 dogs per km^2^ are expected. From this analysis, it was determined that 89.8% of Uganda’s human population (~30 000 000 people) is likely to live in a community that can support enzootic transmission of canine rabies. Approximately 60% of Uganda’s population (26.5 million people) resides in areas where there are greater than ten unvaccinated dogs for every 1 000 people. Interestingly, Knobel et al. in 2005 estimated that 68% of Africans live at risk for rabies; a study which utilized completely different methods and data sources. While this study did not match Knobel’s dog population estimates, there was agreement between Knobel and this study in regards to the large proportion of persons residing in areas of high-risk for rabies transmission [14].

These modelled estimates are meant to provide a proxy measure for the potential rabies activity in a country in which surveillance programs for human and animal cases are not adequate. These modelled estimates should be used to guide decisions on where to allocate rabies control resources and can be used to advocate for more support from the national and international communities. However, these estimates should not be used to replace routine rabies surveillance activities, as surveillance activities are critical both for the treatment of bite victims, monitoring of epidemiological changes, and evaluation of MCV programs. Furthermore, derivation of accurate estimates is an iterative process that should be repeated and refined as additional empirical data are available.

## Conclusions

The results from this study represent some of the most comprehensive data on dog ecology, demographics, and vaccination coverage in Uganda and may be helpful to refine current national and regional rabies burden estimates. The significant association between poverty and dog ownership is likely not unique to Uganda, and other countries should consider exploring this relationship when conducting dog population estimation studies. Furthermore, the findings from this study should be used to enhance current mass canine rabies vaccination strategies in Uganda, through the strategic use of resources where they will have the greatest impact. However, this study has several limitations, including only reflecting the characteristics of the owned dog population. These types of models should always undergo a degree of validation before major programmatic changes are enacted. If evaluation studies are consistent with the findings in this study, canine rabies elimination in Uganda may be more feasible than previously thought. Unfortunately, until successful vaccination strategies are developed and implemented in Uganda, there are likely more than 26 million people that live with the daily risk of becoming exposed to the CRVV from an infected dog. This study provides some guidance on where rabies risks may be highest, and these communities should be engaged to implement rabies prevention activities. Studies which describe the ecology of dogs and characteristics of dog owners are necessary to develop a successful rabies control program and the findings from this study should be considered by national and international programs.

## Figures and Tables

**Fig. 4:**
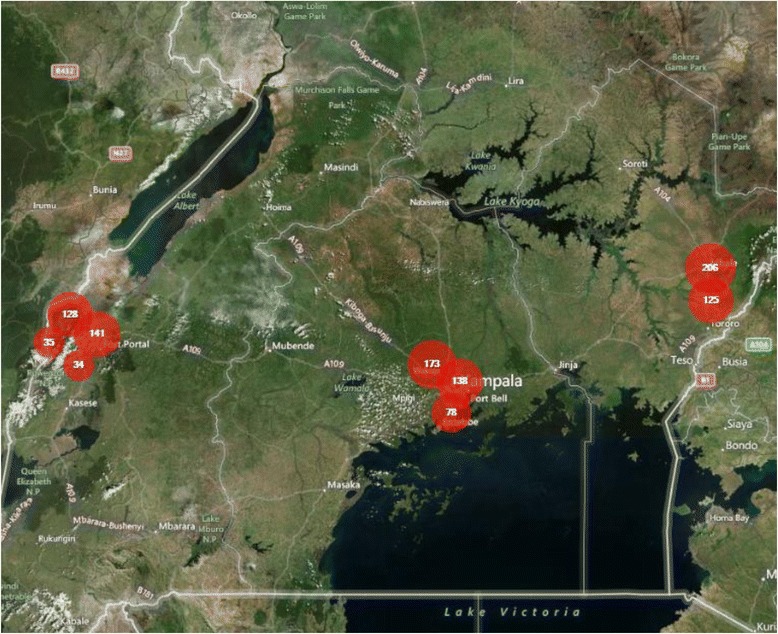
Study locations and community surveys completed in Uganda, 2013

## Appendix 1

### Survey questionnaire

1. Interview Date:
2. Interviewer:
3. Consent obtained (Note: Form requests confirmation of adult age)
  - Yes
  - No
4. How old are you?
5. Gender
  - Male
  - Female
6. How many years of schooling have you completed?
7. How many people live in your household?
8. How many children below the age of 18 live in your household?
9. How many years have you lived in this place?
10. (Surveyor assistant - observe and describe construction of house).
  - Floor – cement/tile/dirt/other:
  - Walls – cement/metal/mud/straw or palm leaves/other:
  - Roof – cement/metal/straw or palm leaves/other:
  - Windows – none/metal/curtain/other:
  - Door – none/metal/curtain/other:
11. What kind of livestock does your family own? How many head of each? Mark all that apply.
  - None
  - Chickens, indicate number
  - Cattle, indicate number
  - Goats, indicate number
  - Sheep, indicate number
  - Other: (free response), indicate number
  - Declined to answer
12. Does your family currently own any dogs? If yes, how many? (**if answer is No, skip to 18**)
  - No
  - Yes, indicate number
  - Declined to answer
13. What are the ages of your dogs?
  - Free response
14. What best describes the amount of time that your dog(s) spends indoors?
  - Never
  - Infrequently
  - Occasionally
  - Frequently
  - Always
  - Declined to answer
15. What level of care do you provide for your dog(s)? Mark all that apply.
  - None
  - Food
  - Water
  - Shelter
  - Veterinary Care
  - Other: (free response)
  - Declined to answer
16. Have any of your dog(s) been vaccinated against rabies?
  - Yes, indicate number
  - No
  - I don’t know
  - Declined to answer
17. If any of your dog(s) have not been vaccinated for rabies, what is the reason?
  - Too young
  - No money to buy vaccine
  - No vaccine available
  - No need to vaccinate
  - Other (free response):
  - Declined to answer
18. In the past five years, have you owned any dogs that died?
  - No
  - Yes, indicate number
  - Declined to answer
19. For the dogs that died, what was the cause of death? Indicate frequency of each if more than one dog.
  - Accident/injury
  - Disease/illness
  - Other: free response
  - I don’t know
  - Declined to answer
20. Does your family care for any dogs in the community? If yes, how many? (**if answer is No, skip to 22**)
  - No
  - Yes, indicate number
  - Declined to answer
21. What level of care do you provide for the community dog(s)? Mark all that apply.
  - None
  - Food
  - Water
  - Shelter
  - Veterinary Care
  - Other: (free response)
  - Declined to answer
22. Have you or anyone in the household been bitten by a dog? Mark all that apply. (**if answer is No, skip to 31**)
  - No
  - Yes, me
  - Yes, an adult family member (indicate number if more than one)
  - Yes, my child (indicate number if more than one)
  - Declined to answer
23. For each person identified, how old were you when you were bitten by the dog?
  - Free response
24. For each person identified, on how many separate occasions were you/they bitten by a dog? Mark all that apply and indicate frequency if multiple persons were identified.
  - One occasion
  - Two occasions
  - Three occasions
  - Four occasions
  - Five occasions
  - More than five occasions
25. For each person identified, where were you/they when you were bitten by the dog? Mark all that apply and indicate frequency if multiple persons were identified.
  - At home
  - Not at home, but within local community
  - Outside of local community
  - Declined to answer
26. For each person identified, what were you doing when you/they were bitten the dog? Mark all that apply and indicate frequency if multiple persons were identified.
  - At home, unprovoked attack by own dog
  - At home, unprovoked attack by community dog
  - Playing with, restraining or feeding the dog
  - Playing with, restraining of feeding puppies of the (bitch) dog
  - Visiting the dog’s home
  - Walking in community, avoiding the dog
  - Herding livestock, avoiding the dog
  - Hunting wild animals, avoiding the dog
  - Playing or recreating outdoors, avoiding the dog
  - Other: (free response)
  - Declined to answer
27. For each person identified, where on your body were you/they bitten by the dog? Mark all that apply and indicate frequency if multiple persons were identified.
  - Head/face
  - Torso/trunk
  - Hands/feet
  - Arm
  - Leg
  - Other: (free response)
  - Declined to answer
28. For each person identified, what did you/they do when bitten by the dog? Mark all that apply and indicate frequency if multiple persons were identified.
  - Nothing
  - Washed wound
  - Consulted with a traditional healer
  - Call a medical doctor
  - Call a veterinarian
  - Actively sought medical treatment at a pharmacy, hospital, clinic or outpost
  - Received rabies post-exposure prophylaxis
  - Isolated the dog for observation
  - Submitted dog for disease testing
  - Killed the dog
  - Killed and ate the dog
  - Other: (Free response)
  - Declined to answer
29. (***If answer to 27 was ‘f’ or ‘g’***) What was the amount of time between when you/they were bitten and medical treatment was sought? Mark all that apply and indicate frequency if multiple persons were identified.
  - < 1 day
  - 1–3 days
  - 4–6 days
  - 1–2 weeks
  - 3–4 weeks
  - 5–8 weeks
  - > 2 months
  - Other: (free text)
  - Declined to answer
30. For each person identified, how familiar were you/they with the dog? Mark all that apply and indicate frequency if multiple persons were identified. **Proceed to 33**.
  - Own (family) dog
  - Neighbor’s dog
  - Dog in community
  - Did not recognize dog
  - Declined to answer
31. (***if never been bitten by a dog***) What would you do if you were bitten by a dog that you recognize or own? Mark all that apply.
  - Nothing
  - Wash wound
  - Consult with a traditional healer
  - Call a medical doctor
  - Call a veterinarian
  - Actively seek medical treatment at a pharmacy, hospital, clinic or outpost
  - Receive rabies post-exposure prophylaxis
  - Isolate the dog for observation
  - Submit dog for disease testing
  - Kill the dog
  - Kill and eat the dog
  - Other: (Free response)
  - Declined to answer
32. (***if never been bitten by a dog***) What would you do if you were bitten by a dog that you do not recognize or own? Mark all that apply.
  - Nothing
  - Wash wound
  - Consult with a traditional healer
  - Call a medical doctor
  - Call a veterinarian
  - Actively seek medical treatment at a pharmacy, hospital, clinic or outpost
  - Receive rabies post-exposure prophylaxis
  - Isolate dog for observation
  - Submit animal for disease testing
  - Kill the dog
  - Kill and eat the dog
  - Other: (Free response)
  - Declined to answer
33. If you saw a dog in your village that looked sick, what would you do? Mark all that apply.
  - Nothing
  - Call local authorities
  - Call a friend
  - Avoid the animal
  - Scare (shoo) animal away
  - Kill the dog
  - Kill and eat the dog
  - Submit the animal for disease testing
  - Other: (Free response)
  - Declined to answer
34. Does your family currently own any cats? If yes, how many?
  - No
  - Yes, indicate number
  - Declined to answer
35. Have you or anyone in this household had illness that was attributed to a pet/livestock animal bite? Mark all that apply. (**if answer is No, skip to 38**)
  - No
  - Yes, me
  - Yes, an adult family member (indicate number if more than one)
  - Yes, my child (indicate number if more than one)
  - Declined to answer
36. If the answer to 35 was yes, what type of animal was it?
  - Dog (indicate number if more than one)
  - Cat (indicate number if more than one)
  - Other: free response (indicate number if more than one)
37. If the answer to 35 was yes, what were the symptoms? Mark all that apply and indicate frequency if multiple persons were identified.
  - Skin rash/discoloration/ infection
  - Unusual bleeding (e.g. from nose/mouth)
  - Hypersalivation
  - Fever
  - Cough
  - Sneezing
  - Runny nose
  - Chest congestion
  - Muscle pain
  - Difficulty breathing
  - Headache
  - Convulsions
  - Altered mental state (dementia)
  - Unconsciousness/coma
  - Muscle weakness/paralysis
  - Vomiting or diarrhea or stomach cramps
  - Miscarriage/stillbirth
  - Death
  - Multiple persons
  - Other: (Free response)
  - Declined to answer
38. Have you or anyone in this household been bitten by a wild animal (including rats)? Mark all that apply. (**if answer is No, skip to 47**)
  - No
  - Yes, me
  - Yes, an adult family member (indicate number if more than one)
  - Yes, my child (indicate number if more than one)
  - Declined to answer
39. For each person identified, how old were you when you were bitten by the wild animal?
  - Free response
40. What kind of wild animal was it? Mark all that apply and indicate frequency if multiple persons were identified.
  - Jackal
  - Hyena
  - Mongoose
  - Honey badger
  - Monkey or other primate
  - Fox
  - Bat
  - Rat
  - Other:
  - I don’t know
  - Declined to answer
41. For each person identified, on how many separate occasions were you/they bitten by a wild animal? Mark all that apply and indicate frequency if multiple persons were identified.
  - One occasion
  - Two occasions
  - Three occasions
  - Four occasions
  - Five occasions
  - More than five occasions
42. For each person identified, where were you/they when bitten by the wild animal? Mark all that apply and indicate frequency if multiple persons were identified.
  - At home
  - Not at home, but within local community
  - Outside of local community
  - Declined to answer
43. For each person identified, what were you doing when you were bitten by the wild animal? Mark all that apply and indicate frequency if multiple persons were identified.
  - In home, the animal entered home
  - Walking in community, avoiding the animal
  - Playing with, restraining or feeding the animal
  - Herding livestock, avoiding the animal
  - Hunting other animals
  - Hunting the animal
  - Playing or recreating outdoors, avoiding the animal
  - Other: (free response)
  - Declined to answer
44. For each person identified, where on your body were you/they bitten by the wild animal? Mark all that apply and indicate frequency if multiple persons were identified.
  - Head/face
  - Torso/trunk
  - Hands/feet
  - Arm
  - Leg
  - Other: (free response)
  - Declined to answer
45. For each person identified, what did you do after you/they were bitten by the wild animal? Mark all that apply and indicate frequency if multiple persons were identified.
  - Nothing
  - Washed wound
  - Consulted with a traditional healer
  - Call a medical doctor
  - Call a veterinarian
  - Actively sought medical treatment at a pharmacy, hospital, clinic or outpost
  - Received rabies post-exposure prophylaxis
  - Isolated the animal for observation
  - Submitted animal for disease testing
  - Killed the animal
  - Killed and ate the animal
  - Other: (Free response)
  - Declined to answer
46. (***If answer to 45 was ‘f’ or ‘g’***) For each person identified, what was the amount of time between when you/they were bitten and when medical treatment was sought? Mark all that apply and indicate frequency if multiple persons were identified.
  - < 1 day
  - 1–3 days
  - 4–6 days
  - 1–2 weeks
  - 3–4 weeks
  - 5–8 weeks
  - > 2 months
  - Other: (free text)
  - Declined to answer
47. (***if never been bitten by a wild animal***) If you were bitten by a wild animal, what would you do? Mark all that apply.
  - Nothing
  - Wash wound
  - Consult with a traditional healer
  - Call a medical doctor
  - Call a veterinarian
  - Actively seek medical treatment at a pharmacy, hospital, clinic or outpost
  - Receive rabies post-exposure prophylaxis
  - Isolate the animal for observation
  - Submit animal for disease testing
  - Kill the animal
  - Kill and eat the animal
  - Other: (Free response)
  - Declined to answer
48. If you saw a wild animal in your village that looked sick, what would you do? Mark all that apply.
  - Nothing
  - Call local authorities
  - Call a friend
  - Avoid the animal
  - Scare (shoo) animal away
  - Kill the animal
  - Kill and eat the animal
  - Submit the animal for disease testing
  - Other: (Free response)
  - Declined to answer
49. Have you or anyone in this household had illness that was attributed to a wild animal bite? (**if answer is No, skip to 52**)
  - No
  - Yes, me
  - Yes, an adult family member (indicate number if more than one)
  - Yes, my child (indicate number if more than one)
  - Declined to answer
50. If answer to 49 was yes, what type of animal was it?
  - Jackal
  - Hyena
  - Mongoose
  - Honey badger
  - Monkey or other primate
  - Fox
  - Bat
  - Rat
  - Other:
  - I don’t know
  - Declined to answer
51. For each person identified, what were the symptoms? Mark all that apply and indicate frequency if multiple persons were identified.
  - Skin rash/discoloration/ infection
  - Unusual bleeding (e.g. from nose/mouth)
  - Hypersalivation
  - Fever
  - Cough
  - Sneezing
  - Runny nose
  - Chest congestion
  - Muscle pain
  - Difficulty breathing
  - Headache
  - Convulsions
  - Altered mental state (dementia)
  - Unconsciousness/coma
  - Muscle weakness/paralysis
  - Vomiting or diarrhea or stomach cramps
  - Miscarriage/stillbirth
  - Death
  - Multiple persons
  - Other: (Free response)
  - Declined to answer
52. How much do you know about a disease called rabies? Note: interviewer must evaluate.
  - I have never heard of rabies
  - Little knowledge (i.e., have heard of rabies/dog disease, but can’t identify transmission routes or severity of disease)
  - Basic understanding (knowledge that rabies is both a highly fatal disease and is transmitted by dog bite)
  - Extensive knowledge (basic understanding plus knowledge of non-bite routes of exposure AND wildlife reservoirs besides dogs without prompting)
  - Declined to answer
53. How severe is the disease called rabies?
  - Mild
  - Somewhat severe
  - Very severe, but possible to recover
  - Very severe, resulting in death
  - I don’t know
  - Declined to answer
54. How do humans get rabies from an infected animal? Mark all that apply.
  - Bite
  - Scratch
  - Observing the animal
  - Touching the animal
  - Contact with blood
  - Contact with saliva
  - Contact with urine/feces
  - Other: (free response)
  - I don’t know
  - Declined to answer
55. What animals can be infected with rabies? Mark all that apply
  - Dogs
  - Cats
  - Livestock (Cattle, sheep, goats, etc.)
  - Poultry (Chickens, ducks, geese, etc.)
  - Horses
  - Jackals
  - Hyenas
  - Mongoose
  - Monkeys or other primate
  - Fox
  - Wild Birds
  - Bats
  - Rodents
  - Other: (free response)
  - I don’t know
  - Declined to answer
56. If you thought that you had an exposure to an animal with rabies, what would you do?
  - Nothing
  - Wash wound
  - Consult with a traditional healer
  - Call a medical doctor
  - Call a veterinarian
  - Actively seek medical treatment at a pharmacy, hospital, clinic or outpost
  - Receive rabies post-exposure prophylaxis
  - Isolate the animal for observation
  - Submit animal for disease testing
  - Kill the animal
  - Kill and eat the animal
  - Other: (Free response)
  - Declined to answer
57. Where do you normally go to receive medical treatment? Mark all that apply.
  - Veterinary clinic
  - Pharmacy
  - Medical Clinic
  - Hospital
  - Traditional Healer
  - Other: (free response)
  - Declined to answer
58. How far do you need to travel to receive medical care at this location? Indicate frequency if multiple locations were identified.
  - <1 km
  - 1-5 km
  - 6-10 km
  - 11-20 km
  - 21-30 km
  - >30 km
  - I don’t know
  - Declined to answer
59. How far away is the location where you could receive rabies vaccination?
  - <1 km
  - 1-5 km
  - 6-10 km
  - 11-20 km
  - 21-30 km
  - >30 km
  - I don’t know
  - Declined to answer
60. Have you or anyone in this household ever received rabies post-exposure prophylaxis? Mark all that apply and indicate frequency if needed (if answer is no, skip to **64**)?
  - Yes, pre-exposure prophylaxis
  - Yes, post-exposure prophylaxis
  - No
  - Declined to answer
61. (**If answer to 60 is ‘a’ or ‘b’**) Why did you or someone in your household receive rabies pre-exposure or post-exposure prophylaxis?
  - Pre exposure – free response: (identify any reason(s) that apply)
  - Post exposure – free response: (identify any reason(s) that apply)
  - Declined to answer
62. (**If answer to 61 is ‘b’**) What elements of post-exposure prophylaxis did you or someone in your household receive? Mark all that apply and ndicate frequency if needed.
  - Rabies vaccine – Indicate number of doses (days) that treatment was administered
  - Rabies immune globulin (serum) Indicate number of doses (days) that treatment was administered (Note: should only be on Day 0)
  - Anti-tetanus serum - Indicate number of doses (days) that treatment was administered (Note: not part of rabies PEP, but may be commonly administered for bite wounds)
  - Other – free response
63. Where would (or did) you go to receive rabies vaccination?
  - Pharmacy
  - Medical clinic
  - Traditional healer
  - Veterinary clinic
  - Hospital
  - Other:
  - Declined to answer
64. What are the primary obstacles for getting medical treatment in your community? Mark all that apply.
  - Lack of facilities to provide treatment
  - Lack of trained personnel at facilities to provide treatment
  - Lack of medicines at facilities for treatment
  - No means of transportation
  - No money to pay for treatment
  - Can’t miss work
  - Other: (free text)
  - I don’t know
  - Declined to answer
65. What do you know about veterinarians? Mark the best answer.
  - Person that provides care to sick or injured animals
  - Person that provides care to sick or injured humans
  - Person that provides care to sick or injured humans and animals
  - Person that provides education about animal health
  - Person that provides education about public health
  - Person that provides education about animal and public health
  - I don’t know or have never heard of a veterinarian

## Appendix 2

**Table 6:** Scoring system for evaluating domicile construction quality

	Construction Quality Score (1 = High Quality/3 = Low Quality)		
Domicile Feature	1	2	3
Floors	Cement or Tile	Wood or Brick	Soil
Walls	Cement of Metal	Mud	Straw or Palm Fronds
Roof	Iron or Metal	Cement	Straw or Palm Fronds
Windows	Glass or Metal	Wood	Curtain Only or No Windows
Doors	Metal	Wood	Curtain Only

## Appendix 4

**Table 7:** Human population by community canine rabies vaccination coverage rates, Uganda 2013

Human Population by Community Canine Rabies Vaccination Coverage Rates, Uganda 2013		
Proportion of Dogs Vaccinated	Human Population	Percent of Population
<10%	1,052,831	3.1%
10–<20%	10,354,362	30.1%
20–<30%	13,236,258	38.5%
30–<40%	3,925,318	11.4%
40–<50%	1,325,869	3.9%
50–<60%	765,022	2.2%
60–<70%	394,716	1.1%
≥70%	3,291,725	9.6%
Total	34,346,101	

## Additional file

Additional file 1: Multilingual abstracts in the five official working languages of the United Nations. (PDF 638 kb)

## Abbreviations

aOR: Adjusted ORs
CRVV: Canine rabies virus variant
H:D ratio: Human to Dog population ratio
KAP: Knowledge attitudes and practices
PDA: Personal digital devices
