# Supplementary material for: The impact of poverty on dog ownership and access to canine rabies vaccination: results from a knowledge, attitudes and practices survey, Uganda 2013
Source: Infect Dis Poverty. 2017 Jun 1;6:97. doi: 10.1186/s40249-017-0306-2 (PMC5452361; doi:10.1186/s40249-017-0306-2)
Supplement: Additional file 1: — Multilingual abstracts in the five official working languages of the United Nations. (PDF 638 kb) [file 40249_2017_306_MOESM1_ESM.pdf]

Translation of the abstract into the five official working languages of the United Nations

أثر الفقر على ملكية كلب والحصول على التلقيح ضد السعار الكلبي: نتائج من مسح المعارف والمواقف والممارسات، أوغندا 2013

ريان ماكلارين والاس، جيسون ميهال، يوشينوري ناكازاوا، سيرجيو ريكوينكو، برنابا باكاموتوماهو، مودوبي أوزينوبي، فيكتور توجوميزيمو، جيسي د. بلانتون، ايمي جيلبرت، جوزيف اما

ملخص

خلفية: داء الكلب هو مرض مهمل على الرغم من كونه مسؤولا عن وفاة الإنسان أكثر من أي مرض حيواني المصدر آخر. عدم وجود رصد كاف لتواجد الكلاب بين إنسان أدى إلى عدم إعطاء هذا الأمر الأولوية الكافية وكثيرا ما يلقى باللوم عليه في هذه المفارقة. وغالبا ما تستخدم أساليب تقدير لوصف عبء داء الكلب عندما لا تتوفر بيانات الرصد، ولكن هذه الأرقام نادرا ما تستند إلى البيانات الخاصة بكل بلد.

الطريقة: في عام 2013 تم إجراء مسح للمعرفة والاتجاهات والممارسات في أوغندا لرصد عدد الكلاب المتواجدة في التجمعات البشرية، والتطعيم ضد داء الكلب، وعوامل الخطر الناجمة من داء الكلب على البشر والتحسن تقديرات عبء داء الكلب في البلاد وعلى المستوى الإقليمي. واستخدم توزيع بواسون وتقنيات الانحدار اللوجستي متعدد المستويات لتقدير التغطية الكاملة لتعداد الكلاب ومعدلات التطعيم.

النتائج: تم اختيار أربعة وعشرين قرية، استكملت 798 أسرة بها بيانات الدراسة، وهو ما يمثل 4375 شخص. تمثل الكلب المقتناة من أسر 12.9٪ من تعداد الكلاب المتواجدة بين السكان، حيث بلغ عدد الكلاب المقتناة 175 كلبا (25 شخصا لكل الكلب). بلغت نسبة التطعيم في الكلاب 55.6٪ من الكلاب المقتناة. الفقر والكثافة السكانية البشرية ارتباطا وثيقا باقتناء كلب، وعندما مثلت في نماذج الانحدار متعدد المستويات، وانخفضت نسبة الإنسان إلى الكلب إلى 47: 1 وانخفضت نسبة التغطية لتطعيم الكلاب ضد داء الكلب على المستوى الوطني حيث تشير التقديرات إلى 36.1٪. وتقدر هذه الدراسة أن هناك 729486 كلبا مقتنى في أوغندا (95٪ CI 719919-739053). قدم عشرة في المئة من المشاركين في الاستطلاع الرعاية لكلاب لا يملكونها، ولكن لم توضع الكلاب الضالة ضمن هذا الإحصاء. وقدرت نسبة 89.8٪ من عدد السكان في أوغندا يعيشون في مجتمع يمكن أن يدعم انتقال داء السعار الكلبي المتوطن بالحيوانات.

الاستنتاجات: هذه الدراسة هي الأولى لإجراء تقييم شامل لتأثير الفقر على اقتناء الكلاب في أفريقيا. وتشير هذه النتائج إلى أن تقدير عدد الكلاب قد لا يكون في سهولة تقدير عدد البشر: نسبة الكلاب، والعوامل مثل الفقر من المرجح أن تؤثر بشدة في اقتناء الكلاب وتطعيمها. وينبغي تأكيد هذه التقديرات المنمذجة من خلال المزيد من الدراسات الميدانية، ومع ذلك، إذا تم التحقق من صحتها، فإن القضاء على داء الكلب من خلال التطعيم الشامل قد لا يكون أمرا صعبا كما كان يعتبر في السابق في أوغندا. وينبغي النظر في البيانات المستمدة من هذه الدراسة لتحسين نماذج التقدير لعبء داء الكلب داخل الدولة وعلى المستوى الإقليمي.

Translated from English version into Arabic by Mahmoud Sami, through

TRANSLATORS
WITHOUT BORDERS

贫困对养犬和犬的狂犬病疫苗接种的影响：2013 年乌干达一项知识、态度和行为调查的结果

Ryan MacLaren Wallace, Jason Mehal, Yoshinori Nakazawa, Sergio Recuenco, Barnabas Bakamutumaho, Modupe Osinubi, Victor Tugumizemu, Jesse D. Blanton, Amy Gilbert, Joseph Wama

摘要
引言：尽管所致人类死亡比任何其他一种人兽共病都要多，但是狂犬病仍然是一种被忽视的热带病。由于缺乏足够的人、犬监测导致狂犬病没有获得重视，从而导致了前述的困境。由于缺乏监测数据，因此通常采用估算的方法来描述狂犬病的疾病负担，但是这些数据通常都不是基于特定国家的数据。
方法：2013 年在乌干达开展了一项知识、态度和行为的调查，以便掌握犬数量、狂犬病免疫、人狂犬病危险因素并提高国家和地区的狂犬病疾病负担估计。采用 Poisson 和多水平 logistic 回归模型估计总的犬数量和免疫覆盖情况。
结果：抽取了 24 个村，包括 798 户和 4 375 人。养犬家庭占总人数的 12.9%，养犬数量为 175 只，即每 25 人拥有一只犬。55.6%的饲养犬既往接种过疫苗。贫困和人口密度与养犬高度相关，当采用多水平回归模型以考虑上述因素后，人犬比例降到 47:1，而全国的犬狂犬病接种覆盖率降到 36.1%。本研究估计乌干达全国有 729 486 只犬（95% 置信区间： 719 919-739 053）。调查中 10%的应答者曾经为流浪犬提供过照料，但是流浪犬并没有纳入上述估算中。89.8%的乌干达人居住在地方性狂犬病传播地区。
结论：本研究第一次在非洲全面地评估了贫困对于养犬的影响。研究表明分析犬种数量不比采用人犬比分析简单，贫困等因素很可能对于犬养殖和免疫接种有非常严重的影响。该模型估算的结果需要进一步通过现场研究证实。如果被证实的话，那么通过大规模免疫来消除乌干达的狂犬病就没有先前认为的那么困难。本研究获得的数据可考虑用于改进估算国家和地区的狂犬病疾病负担的模型中。

Translated from English version into Chinese by Men-Bao Qian

CDC CHINESE CENTER FOR DISEASE CONTROL AND PREVENTION
NATIONAL INSTITUTE OF PARASITIC DISEASES

Impact de la pauvreté sur la possession de chiens et leur vaccination antirabique : résultats d'une enquête sur les connaissances, les attitudes et les pratiques en Ouganda en 2013

Ryan MacLaren Wallace, Jason Mehal, Yoshinori Nakazawa, Sergio Recuenco, Barnabas Bakamutumaho, Modupe Osinubi, Victor Tugumizemu, Jesse D. Blanton, Amy Gilbert, Joseph Wama

RÉSUMÉ
Contexte : Bien qu'elle cause plus de décès humains que toute autre zoonose, la rage est une maladie négligée. On explique souvent ce paradoxe par l'absence d'une surveillance adéquate des humains et des chiens, dont la conséquence est qu'elle ne compte pas au rang des priorités. Le fardeau de la rage est souvent estimé lorsqu'il n'existe pas de données de surveillance, mais ces chiffres s'appuient rarement sur des données spécifiques par pays.
Méthodes : Nous avons mené en 2013 en Ouganda une enquête sur les connaissances, les attitudes et les pratiques dont le but était de comprendre les facteurs de risque liés à la population canine, à la vaccination antirabique et à la rage humaine et d'améliorer les estimations nationales et régionales du poids de la rage. Des méthodes de régression logistique multiniveaux et de Poisson ont été appliquées pour estimer la population canine totale et la couverture vaccinale.

Résultats : Nous avons sélectionné 24 villages dans lesquels 798 foyers, représentant 4375 personnes, ont répondu à l'enquête. Les foyers possédant des chiens représentaient 12,9 % de la population et possédaient 175 chiens (25 humains pour 1 chien). Sur les chiens possédés par les foyers, 55,6 % avaient été vaccinés. La pauvreté et la densité de population humaine étaient fortement corrélées à la possession de chiens. En les prenant en compte dans des modèles de régression multiniveaux, nous avons vu baisser le rapport entre humains et chiens à 47 pour 1 et la couverture nationale estimée de la vaccination antirabique canine à 36,1 %. Cette étude estime qu'il y a 729 486 chiens appartenant à des foyers en Ouganda (IC à 95 % de 719 919 à 739 053). Dix pour cent des personnes ayant répondu à l'enquête s'occupaient de chiens qui n'étaient pas à elles mais notre estimation n'a pas tenu compte des chiens errants. Nous avons estimé que 89,8 % de la population humaine de l'Ouganda vivait dans une communauté où la transmission enzootique de la rage canine était favorisée.
Conclusions : Cette étude est la première à évaluer en détail l'effet de la pauvreté sur la possession de chiens en Afrique. Ses résultats indiquent que la description de la population canine peut être plus complexe qu'un simple ratio humains-chiens et que des facteurs tels que la pauvreté peuvent fortement influer sur la possession de chiens et la couverture vaccinale. Ces estimations modélisées restent à confirmer par de nouvelles études sur le terrain. Si elles sont validées, l'élimination de la rage canine par une vaccination de masse en Ouganda pourrait être moins difficile qu'on ne le pensait jusqu'ici. Il serait judicieux de prendre en compte les données tirées de cette étude pour améliorer les modèles d'estimation du fardeau de la rage à l'échelle du pays et de la région.

Translated from English version into French by Suzanne Assenat, through

Воздействие бедности на владение собаками и доступ к прививкам против собачьего бешенства: результаты обследования осведомлённости, мнений и обычаев, Уганда, 2013 г.

Райан Мак-Ларен Уоллес, Джейсон Мейал Йошинори Наказава, Серджо Рекуэнко, Барнабас Бакамутумао, Модупе Осинуби, Виктор Тугумизему, Джесси Д. Блентон, Эйми Джилберт, Джозеф Уама.

РЕФЕРАТ

История вопроса: Бешенство можно отнести к забытым заболеваниям, несмотря на то, что оно вызывает больше человеческих смертей, чем любая другая болезнь, передаваемая от животных человеку. Причина этого парадокса заключается в недостатке надлежащего наблюдения за животными и людьми, ведущем к низкой приоритизации. Для описания заболеваемости бешенством при отсутствии данных наблюдений часто используются оценочные методы, однако результаты редко основываются на данных, относящихся к конкретной стране.

Методы: В 2013 г. в Уганде было произведено обследование осведомлённости, мнений и обычаев с целью оценки размеров популяции собак, охвата прививками против бешенства и факторов риска заболеваемости людей и повышения точности оценки бремени бешенства на национальном и региональном уровне. Для оценки общих размеров популяции собак и ее охвата вакцинацией использовались распределение Пуассона и многоуровневый логистический регрессивный анализ.

Результаты: Было отобрано двадцать четыре деревни, в которых 798 подворий, представляющих 4375 человек, приняло участие в обследовании. Домашние хозяйства, владеющие собаками, составили 12,9%, что означает 175 собак (25 человек на собаку). В 55,6% случаев находящихся в собственности собак сообщалось о выполненных прививках. Отмечена высокая корреляция между уровнем бедности и плотностью населения и наличием собак в собственности в домашних хозяйствах. При учете в многоуровневом логистическом регрессивном анализе соотношение между людьми и собаками упало до 47:1 а расчетный охват прививками против бешенства на национальном уровне - до 36,1%. По оценкам данного исследования в Уганде имеется 729486 собак, имеющих хозяина (95% *CI* (доверительный интервал при доверительной вероятности) 719 919 – 739 053). Десять процентов от участвующих в опросе заботились о собаках, которые им не принадлежали, однако в этом исследовании не учитывались популяции бродячих собак. 89,8% населения Уганды проживает в общинах, в которых возможна энзоотическая передача собачьего бешенства.

Выводы: Данное исследование впервые дало полномасштабную оценку воздействия бедности на владение собаками в Африке. Эти результаты показывают, что описание популяции собак гораздо сложнее, чем применение соотношения размеров населения и количества собак, а уровень бедности оказывает серьезное влияние на владение собаками и на охват вакцинацией. Данная смоделированная оценка должна быть подтверждена полевыми исследованиями со сбором фактических данных на местах, однако такое подтверждение будет означать, что уничтожение собачьего бешенства в Уганде путем массовой вакцинации может оказаться гораздо проще, чем это считалось ранее. Данные, полученные в результате этого исследования, должны использоваться для усовершенствования моделей оценки бремени бешенства на национальном и региональном уровне.

Translated from English version into Russian by Alena Hrybouskaya through

TRANSLATORS
WITHOUT BORDERS

El impacto de la pobreza sobre la tenencia de perros y el acceso a las vacunas antirrábicas caninas: resultados de la encuesta sobre conocimientos, actitudes y prácticas, Uganda 2013

Ryan MacLaren Wallace, Jason Mehal, Yoshinori Nakazawa, Sergio Recuenco, Barnabas Bakamutumaho, Modupe Osinubi, Victor Tugumizemu, Jesse D. Blanton, Amy Gilbert, Joseph Wama

RESUMEN

Antecedentes: la rabia es una enfermedad desatendida pese a ser responsable de causar más muertes humanas que cualquier otra zoonosis. A menudo se culpa de esta paradoja a la falta de adecuados controles humanos y caninos, consecuencia de la baja prioridad que se le asigna. Con frecuencia se utilizan métodos de estimación para describir la carga de la rabia cuando no se dispone de datos de control; no obstante, estas cifras rara vez se basan en datos específicos de un país.

Métodos: en 2013, se llevó a cabo la encuesta sobre conocimientos, actitudes y prácticas en Uganda para comprender la población canina, la vacuna antirrábica y los factores de riesgo de contraer rabia humana, y para mejorar las estimaciones de la carga de la rabia a nivel regional y dentro del país. Se utilizaron técnicas de regresión logística de varios niveles y de Poisson para estimar la población canina total y la cobertura de las vacunas.

Resultados: se seleccionaron veinticuatro aldeas. En ellas, 798 familias completaron la encuesta, lo que representaba una cantidad de 4.375 personas. Las familias que tenían perros representaban el 12,9 % de la población, 175 de los perros tenían dueño (25 personas por perro). Se registraron antecedentes de vacunación en el 55,6 % de los perros con dueño. La densidad demográfica y la pobreza estaban estrechamente relacionadas con la tenencia de perros y cuando se las tomó en cuenta en modelos de regresión de varios niveles, la relación de seres humanos respecto a perros registraba una baja de 47:1 y la cobertura nacional estimada de vacunación antirrábica canina había disminuido al 36,1 %. Este estudio estima que hay 729.486 perros con dueño en Uganda (IC del 95 % 719.919 – 739.053). El diez por ciento de las personas encuestadas cuidaba a perros que no eran de su propiedad; sin embargo, las poblaciones de perros callejeros no fueron contabilizadas en esta estimación. Se estimó que el 89,8 % de la población humana de Uganda reside en una comunidad que puede resistir la transmisión de la rabia canina enzoótica.

Conclusiones: este estudio es el primero en evaluar, de forma exhaustiva, el efecto de la pobreza en la tenencia de perros en África. Estos resultados indican que describir una población canina podría no ser tan sencillo como utilizar la proporción ser humano:perro y que es posible que factores tales como la pobreza ejerzan gran influencia en la tenencia de perros y en la cobertura de vacunación. Deben hacerse estudios de campo adicionales para confirmar estas estimaciones modeladas; no obstante, si se validaran, la eliminación de la rabia canina a través de la vacunación masiva podría resultar menos dificultosa de lo que se pensaba previamente en Uganda. Los datos obtenidos en este estudio deben tenerse en cuenta para mejorar los modelos utilizados para las estimaciones de la carga de la rabia a nivel regional y dentro del país.

Translated from English version into Spanish by dsp3112, through

TRANSLATORS
WITHOUT BORDERS
